# Diagnostic and Prognostic Value of AISI, SII, and SIRI in Predicting Gangrenous Evolution of Acute Lithiasic Cholecystitis

**DOI:** 10.3390/diagnostics16030441

**Published:** 2026-02-01

**Authors:** Catalin Vladut Ionut Feier, Melania Veronica Ardelean, Vasile Gaborean, Calin Muntean, Alaviana Monique Faur, Vladut Iosif Rus, Beniamin Sorin Dragan, Marius Sorin Murariu

**Affiliations:** 1 Abdominal Surgery and Phlebology Research Center, “Victor Babeş” University of Medicine and Pharmacy Timişoara, Eftimie Murgu Square No. 2, 300041 Timişoara, Romania; catalin.feier@umft.ro (C.V.I.F.);; 2 First Surgery Clinic, “Pius Brinzeu” Clinical Emergency Hospital, 300723 Timişoara, Romania; 3 Department V, Internal Medicine I, “Victor Babeş” University of Medicine and Pharmacy Timişoara, Eftimie Murgu Square No. 2, 300041 Timişoara, Romania; 4 Thoracic Surgery Research Center, “Victor Babeş” University of Medicine and Pharmacy Timişoara, Eftimie Murgu Square No. 2, 300041 Timişoara, Romania; 5 Department of Surgical Semiology, “Victor Babeş” University of Medicine and Pharmacy Timişoara, Eftimie Murgu Square No. 2, 300041 Timişoara, Romania; 6 Medical Informatics and Biostatistics, Department III-Functional Sciences, “Victor Babeş” University of Medicine and Pharmacy Timişoara, Eftimie Murgu Square No. 2, 300041 Timişoara, Romania; cmuntean@umft.ro; 7 Department of Doctoral Studies, “Victor Babeş” University of Medicine and Pharmacy Timişoara, Eftimie Murgu Square No. 2, 300041 Timişoara, Romania; alaviana.faur@umft.ro; 8 Faculty of Medicine, “Victor Babeş” University of Medicine and Pharmacy Timişoara, Eftimie Murgu Square No. 2, 300041 Timişoara, Romania

**Keywords:** gangrenous cholecystitis, AISI (aggregate index of systemic inflammation), SII (systemic immune-inflammation index), SIRI (systemic inflammation response index), inflammation biomarkers, preoperative risk stratification, predictive hematological indices

## Abstract

**Background/Objectives:** Acute calculous cholecystitis remains one of the most frequent surgical emergencies, ranging from mild inflammation to gangrenous forms associated with necrosis and sepsis. Early differentiation between these stages is essential for timely surgical management. This study aimed to evaluate the diagnostic and prognostic value of hematological inflammatory indices—the Aggregate Index of Systemic Inflammation (AISI), the Systemic Immune-Inflammation Index (SII), and the Systemic Inflammation Response Index (SIRI)—in predicting the gangrenous evolution of acute calculous cholecystitis. **Methods:** A retrospective study was conducted on 435 patients who underwent cholecystectomy between 2016 and 2024 at a tertiary care center. Patients were divided into gangrenous (*n* = 145) and chronic (*n* = 290) cholecystitis groups. Preoperative hematological parameters were used to calculate AISI, SII, and SIRI. After histopathological confirmation, patients with GC (gangrenous calculous cholecystitis) were identified, and for each case, two age- and sex-matched controls with chronic CC (calculous cholecystitis) were selected, maintaining a GC:CC ratio of 1:2. Preoperative hematological parameters were used to calculate AISI, SII, and SIRI. **Results:** All three indices were significantly higher in the gangrenous group (*p* < 0.001). Logistic regression identified SIRI as the strongest independent predictor of gangrenous cholecystitis (OR = 1.976, *p* < 0.001). ROC analysis demonstrated excellent discriminative capacity for all markers (AUC > 0.8), with SII achieving the highest diagnostic accuracy (AUC = 0.889, sensitivity 79.5%, specificity 86.3%). **Conclusions:** AISI, SII, and SIRI represent reliable, easily obtainable, and noninvasive biomarkers for assessing inflammatory severity and predicting gangrenous transformation in acute calculous cholecystitis. Their integration into preoperative evaluation could improve early risk stratification, surgical planning, and patient outcomes.

## 1. Introduction

Acute calculous cholecystitis represents one of the most frequent surgical emergencies of the digestive tract, being primarily caused by cystic duct obstruction due to gallstones [1,2,3]. Its clinical course start from mild, self-limiting inflammation to severe forms such as GC (gangrenous cholecystitis), which is associated with mural necrosis, perforation, and an increased risk of sepsis [4,5]. Whereas, CC (chronic calculous cholecystitis) results from a sustained inflammatory process characterized by recurrent subclinical episodes, progressive fibrosis, and thickening of the gallbladder wall [6,7,8].

Early and precise differentiation among the clinical forms of acute cholecystitis is essential for optimizing patient outcomes, as gangrenous transformation may develop swiftly and is associated with substantially increased morbidity and mortality in cases of delayed surgical management [9,10]. Pathophysiologically, cystic duct obstruction triggers bile stasis, ischemia, and an inflammatory cascade mediated by neutrophil infiltration, cytokine release, and microcirculatory impairment [11,12]. The magnitude of this response determines disease severity, yet routine markers such as leukocytosis and C-reactive protein (CRP) often fail to distinguish gangrenous from uncomplicated cases [13,14].

In this context, several composite indices derived from the complete blood count have been proposed, reflecting in a comprehensive manner the balance between inflammatory and immune responses [15]. They are: Systemic Immune-Inflammation Index (SII), the Systemic Inflammation Response Index (SIRI), and the Aggregate Index of Systemic Inflammation (AISI). These blood-derived markers provide an integrated perspective on systemic inflammatory status, illustrating the interaction between innate and adaptive immune responses [16,17,18].

Neutrophils, monocytes, lymphocytes, and platelets act in close coordination during the inflammatory response, shaping both its intensity and outcome. Neutrophils represent the earliest responders, eliminating pathogens through phagocytosis and the release of lysosomal enzymes and reactive oxygen species—processes particularly accentuated in bacterial infections and ischemic–necrotic injuries such as gangrenous cholecystitis [19,20,21]. Monocytes and their macrophage derivatives sustain inflammation through the secretion of cytokines including IL-1β, TNF-α, and IL-6, while also contributing to subsequent tissue repair [22,23]. Conversely, lymphocyte depletion reflects impairment of the adaptive immune system and has been associated with oxidative stress and systemic inflammatory imbalance [21,24]. Platelets, traditionally linked to coagulation, also modulate inflammation by releasing mediators such as platelet factor-4, serotonin, and thromboxane A_2_, which enhance leukocyte recruitment [20,23].

On this basis, composite indices derived from these parameters—SII = (Neutrophils × Platelets)/Lymphocytes, SIRI = (Neutrophils × Monocytes)/Lymphocytes, and AISI = (Neutrophils × Monocytes × Platelets)/Lymphocytes—provide a quantitative overview of the inflammatory–immune balance [16,17,18,25]. Elevated values of these indices indicate an intensified inflammatory state, microcirculatory impairment, and a higher likelihood of disease progression toward complicated or gangrenous cholecystitis [12,17,18,26,27,28].

Gangrenous cholecystitis is frequently accompanied by challenging intraoperative conditions, a greater likelihood of conversion to open surgery, and an increased incidence of postoperative complications. Previous studies on laparoscopic cholecystectomy have highlighted that the degree of systemic inflammation represents a major determinant of operative complexity, with elevated inflammatory indices often preceding technically demanding surgical procedures [3,10]. Moreover, severe or gangrenous forms of the disease have occasionally been reported in pediatric and adolescent patients, in whom surgical indications tend to be more restrictive and cumulative operative experience with this pathology is limited compared with adult populations. These factors may contribute to delayed intervention and a correspondingly higher risk of adverse outcomes in younger patients [29].

Based on these data, assessment of AISI, SII, and SIRI gains particular importance in surgical practice, providing an accessible, non-invasive, and reproducible method for differentiating CC from GC. An integrative analysis of these parameters may offer essential information for determining disease severity, predicting complications, and optimizing the surgical timing [17,25,30].

In this context, the present study aims to evaluate the predictive value of the composite inflammatory indices AISI, SII, and SIRI in differentiating acute gangrenous calculous cholecystitis from chronic calculous cholecystitis in a cohort of patients operated in a tertiary center. By correlating these indices with clinical and histopathological parameters, the study seeks to define diagnostic thresholds that can be applied in routine practice for the early identification of severe disease forms and for guiding optimal surgical management.

## 2. Materials and Methods

### 2.1. Study Design and Patient Selection

This retrospective study included patients who underwent cholecystectomy at the First Surgery Clinic of the Pius Brînzeu County Emergency Clinical Hospital in Timișoara, Romania, a tertiary care institution. Medical records were reviewed for procedures performed between 1 January 2016, and 31 December 2024.

Specific inclusion criteria were defined to ensure consistency within the study cohort. Eligible cases consisted of patients who had undergone surgical treatment for gangrenous or chronic calculous cholecystitis during the study period. Only individuals aged 18 years or older were included. Considering the well-documented influence of neoplastic diseases on systemic inflammation [31,32], patients with active cancer, a history of malignancy, or prior oncologic therapies (chemotherapy, radiotherapy, or immunotherapy) were excluded.

After reviewing the postoperative histopathology reports, patients diagnosed with gangrenous calculous cholecystitis were identified. Patients with GC were matched with controls diagnosed with CC in a 1:2 ratio using exact matching by sex and age (±2 years) to ensure demographic comparability between groups. CC served as the control histopathological group, selected for its diagnostic homogeneity and reproducibility, rather than as an acute comparator.

After applying the inclusion criteria, data from 435 patients were included in the final statistical analysis.

### 2.2. Data Collection and Variables

Demographic variables such as age, gender, and place of residence (urban/rural) were recorded for all participants. Preoperative hematological parameters were obtained at the time of hospital admission, prior to surgical intervention, from routine complete blood count analyses, and included the following:

- Lymphocytes (Lym);
- Monocytes (Mon);
- Neutrophils (Neu);
- Platelets (Pla).

From these values, several inflammation-based indices were calculated:

- AISI (Aggregate Index of Systemic Inflammation) = (Neu × Mon × Pla)/Lym;
- SIRI (Systemic Inflammation Response Index) = (Neu × Mon)/Lym;
- SII (Systemic Immune-Inflammation Index) = (Neu × Pla)/Lym.

Clinical variables were also recorded, including the presence of nausea, vomiting, fever, and abdominal pain at admission. The type of surgical approach (laparoscopic vs. open cholecystectomy) and the need for conversion from laparoscopic to open surgery were also analyzed.

Histopathological examination of the surgical specimens classified patients into two diagnostic categories:

- Gangrenous cholecystitis (GC);
- Chronic cholecystitis (CC).

Gangrenous cholecystitis was defined based on postoperative histopathological examination by the presence of mural necrosis, ischemic changes, or transmural inflammation, while chronic cholecystitis was defined by postoperative histopathological findings of chronic inflammatory infiltrate, fibrosis, and gallbladder wall thickening.

The main objective of this study was to determine how effectively hematologic inflammation-based indices AISI, SII, and SIRI—could predict the presence of GC in patients who underwent cholecystectomy, with confirmation established through histopathological evaluation.

In addition, the study explored several secondary aspects: first, the diagnostic performance of AISI, SII, and SIRI was examined using receiver operating characteristic (ROC) curve analysis to establish their optimal cut-off values. The predictive performance of these indices was further evaluated through binary logistic regression models to determine their independent association with GC. Secondly, possible relationships between these indices and intraoperative variables—such as the surgical approach (laparoscopic versus open) and the rate of conversion—were investigated to assess their relevance in operative planning.

### 2.3. Ethical Considerations

The study was conducted in accordance with the ethical principles of the Declaration of Helsinki and was approved by the Ethics Committee of the “Pius Brînzeu” County Emergency Clinical Hospital, Timișoara, Romania (Approval No. 551/23 June 2025). According to institutional policy and national regulations, the requirement for informed consent was waived, as the study involved anonymized, retrospectively collected data.

### 2.4. Statistical Analysis

The Shapiro–Wilk test was used to assess the normality of numerical variables. Continuous variables were summarized using descriptive statistics—mean (M) and standard deviation (SD)—while categorical variables were presented as frequencies and percentages.

Comparisons between the two study groups were performed using the Mann–Whitney U test for continuous variables not normally distributed. For categorical variables, the Chi-square test or Fisher’s exact test was applied as appropriate.

To minimize potential collinearity among the indices, three separate logistic regression models were built, each including a single inflammatory marker (AISI, SII, or SIRI) as the independent variable. Odds ratios (OR) with 95% confidence intervals were calculated to evaluate the predictive strength of each index.

The diagnostic performance of each index was assessed using receiver operating characteristic (ROC) curve analysis, with the area under the curve (AUC) calculated for each marker. Youden’s Index was applied to determine optimal cut-off values, and corresponding sensitivity, specificity, and likelihood ratios were reported.

A *p*-value < 0.05 was considered statistically significant. All statistical computations were performed using Jamovi software (version 2.6.26.0), and graphs were generated using Microsoft Excel and Jamovi.

Generative artificial intelligence (GenAI) has been used in this paper to exclusively improve the manuscript’s language and readability. All the scientific content, interpretations, and conclusions are the original work of the authors.

## 3. Results

Data from a total of 435 patients who underwent cholecystectomy at the First Clinic of General Surgery, Pius Brînzeu County Emergency Clinical Hospital, Timișoara, between 1 January 2016, and 31 December 2024, were taken into consideration for this study.

### 3.1. General Aspects

Of the 435 patients, 390 (90.3%) underwent laparoscopic cholecystectomy, 27 (6.3%) were operated using the open surgical approach, and in 15 cases (3.5%), conversion from laparoscopy to the open technique was required.

The open approach was significantly more frequent among patients diagnosed with GC compared to those with the chronic form (10.5% vs. 4.2%, *p* = 0.029). Similarly, the rate of conversion from laparoscopic to open surgery was also higher in the GC group (5.55% vs. 3.1%, *p* = 0.029).

The demographic characteristics of these patients are summarized in Table 1.

### 3.2. Signs and Symptoms

The symptoms and clinical signs reported at presentation are summarized in Table 2.

The variation in inflammatory markers between the two patient groups is presented in Table 3.

Regarding the presence of nausea at admission, no significant differences were found in terms of patient age between those who presented with this symptom and those who did not (62.68 ± 12.66 years vs. 61.34 ± 14.00 years, *p* = 0.347). However, nausea was observed significantly more frequently among female patients compared to males (78.6% vs. 68.8%, *p* = 0.02).

Among the investigated inflammatory status parameters, significant differences were noted for all markers except for total lymphocyte count (1.88 [1.40 to 2.48] vs. 1.77 [1.38 to 2.34], *p* = 0.362), while the remaining parameters did not show statistically significant variations.

The detailed results are summarized in Table 4.

Regarding the presence of vomiting at admission, no significant difference was observed in the mean age between patients who presented with this symptom and those who did not (62.39 ± 13.05 years vs. 62.7 ± 13.02 years, *p* = 0.920). However, vomiting occurred significantly more frequently among female patients compared to males (59.7% vs. 52.1%, *p* = 0.042).

Among the inflammatory status parameters, significant differences were observed in neutrophil count (5.70 [3.82 to 9.54] vs. 4.45 [3.54 to 5.80], *p* < 0.001), platelet count (257 [215 to 308] vs. 240 [201 to 295], *p* = 0.032), AISI (335 [194 to 740] vs. 268 [158 to 501], *p* = 0.013), and SII (791 [467 to 1758] vs. 595 [419 to 917], *p* < 0.001), while the remaining parameters showed no statistically significant differences.

Regarding fever at admission, there were no significant differences in age between patients who presented with fever and those who did not (61.32 ± 14.14 years vs. 62.43 ± 12.92 years, *p* = 0.621). Similarly, fever was not more frequent in female patients compared with males (51.4% vs. 49.6%, *p* = 0.606).

Among the evaluated inflammatory markers, no significant differences were found in lymphocyte (*p* = 0.054), monocyte (*p* = 0.257), or platelet counts (*p* = 0.699). However, statistically significant differences were observed in neutrophil counts (7.94 [4.07 to 12.8] vs. 4.71 [3.62 to 7.41], *p* = 0.001), as well as in AISI (480 [198 to 1406] vs. 294 [178 to 596], *p* < 0.001), SII (1268 [533 to 2910] vs. 664 [439 to 1247], *p* < 0.001), and SIRI (1.71 [0.89 to 5.06] vs. 1.21 [0.74 to 2.27], *p* < 0.001).

### 3.3. Predictive and Diagnostic Value of AISI, SII, and SIRI in Gangrenous Cholecystitis

Given the significant differences observed between groups, a more comprehensive analysis was performed to identify independent markers associated with the GC.

To determine the independent predictive value of the composite inflammatory indices, a binary logistic regression analysis was conducted, with the presence of GC as the dependent variable and AISI, SII, and SIRI scores as independent variables.

In separate binary logistic regression models, all three indices (AISI, SII, and SIRI) were independently associated with gangrenous cholecystitis (*p* < 0.001 for each). SIRI exhibited the strongest predictive power (OR = 1.976, 95% CI 1.62–2.41), indicating that each unit increase nearly doubled the odds of gangrenous evolution. SII (OR = 1.002, 95% CI 1.001–1.003) and AISI (OR = 1.002, 95% CI 1.001–1.003) also showed significant associations, albeit with smaller effect sizes.

The detailed results are presented in Table 5, Table 6 and Table 7.

**Figure 1:**
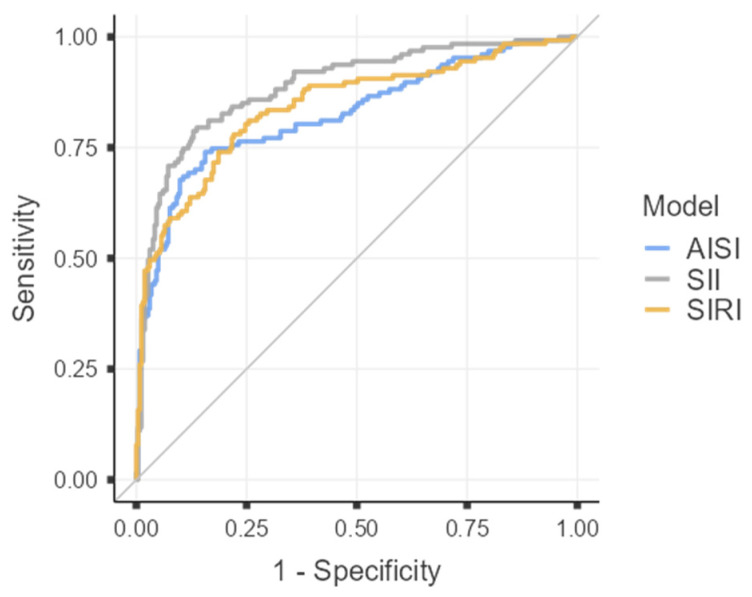
Roc curves for AISI. SII, SIRI.

To assess the discriminative ability of each inflammatory index in differentiating acute gangrenous from chronic forms of cholecystitis, receiver operating characteristic (ROC) curves were constructed for the three analyzed parameters (AISI, SII, and SIRI), as illustrated in Figure 1, and Table 8.

Given the excellent discriminative capacity of all three indices (AUC > 0.8), diagnostic threshold values were determined by calculating the optimal cut-off points using Youden’s Index.

Among the analyzed parameters, SII demonstrated the highest diagnostic performance in differentiating GC from CC, with a cut-off value of 975.75, sensitivity of 79.53%, specificity of 86.31%, and an overall accuracy of 84.10%.

For AISI, the optimal threshold was 431.73, corresponding to a sensitivity of 74.02%, specificity of 84.41%, and an overall accuracy of 81.03%.

In comparison, SIRI had a cut-off value of 1.49, with both sensitivity and specificity of 77.95%, resulting in an overall accuracy of 77.95%.

## 4. Discussion

The findings of this study underscore the pivotal role of hematologically derived inflammatory indices (AISI, SII, and SIRI) in delineating the severity of the inflammatory response in acute calculous cholecystitis, thereby corroborating recent evidence reported in the international literature [10,33]. In an extensive cohort of 435 patients, of whom 145 presented with the gangrenous form and 290 with the chronic form, significant intergroup differences were identified for all investigated hematological markers, supporting the presence of an intensified systemic inflammatory response and immune imbalance characteristic of severe disease.

The classical clinical signs and symptoms—abdominal pain, nausea, vomiting, and fever—were significantly more frequent in patients with GC (*p* < 0.001 for vomiting and fever). These findings are consistent with previous reports in the literature, which have shown that the intensity of clinical manifestations reflects the severity of systemic inflammation and the extent of mural necrosis [1,34].

The analysis of symptomatology also suggests the existence of sex-related differences in immune response. The higher frequency of nausea and vomiting in female patients (*p* = 0.02 and *p* = 0.042) may be associated with hormone-mediated variations in the inflammatory response and an increased susceptibility to cholelithiasis [35,36]. However, these variations did not influence the fundamental hematological parameters, confirming that disease severity is primarily determined by the intensity of inflammation rather than demographic factors [37].

### 4.1. Hematological Changes

Compared with chronic cases, gangrenous cholecystitis was characterized by elevated neutrophil, monocyte, and platelet counts and reduced lymphocytes, reflecting systemic inflammatory activation with suppression of the adaptive response, a mechanism also noted in other acute intra-abdominal infections [4,13].

Elevated mean values of SII, SIRI, and AISI in the gangrenous group (*p* < 0.001 for all) further confirm their role as reliable indicators of inflammatory severity. Notably, SII and SIRI demonstrated pronounced differences between chronic and gangrenous forms of calculous cholecystitis. Median SII values were 1849 [1077 to 2731] in patients with gangrenous cholecystitis (GC) compared with 530 [392 to 743] in those with chronic calculous cholecystitis (CC) (*p* < 0.001), while median SIRI values were 3.03 [1.55 to 7.14] versus 0.94 [0.62 to 1.41] (*p* < 0.001), respectively. These results are consistent with findings from other studies, who reported SII values above 900–1000 as indicators of severe inflammatory forms [10,38].

The monocytosis observed in GC patients 0.48 [0.25 to 0.91] compared with 0.43 [0.30 to 0.60] in chronic cases (*p* < 0.001), indicates persistent activation of the chronic inflammatory phase and direct involvement in the secretion of IL-1β and TNF-α [39,40]. Meanwhile, marked neutrophilia (9.46 [6.60 to 13.4] vs. 4.18 [3.29 to 5.17], *p* < 0.001) reinforces the hypothesis of an ischemic-necrotic inflammatory response, while moderate thrombocytosis (*p* = 0.032) complements the overall profile of systemic inflammatory activation [41,42].

### 4.2. Clinical–Biological Associations

Comparative analyses of symptomatic subgroups provided additional insights into the prognostic value of inflammatory markers. In patients presenting with nausea and vomiting, elevated levels of SII, SIRI, and AISI were associated with greater inflammatory intensity and likely with impaired gallbladder microcirculation [1,4,10]. Nausea was significantly more frequent in patients with high AISI values (*p* = 0.021), whereas vomiting was associated with marked neutrophilia and thrombocytosis (*p* < 0.001 and *p* = 0.029, respectively). Additionally, the presence of fever correlated with markedly elevated SIRI and AISI values (1.29 [0.76 to 2.64] versus 1.11 [0.69 to 1.65] (*p* = 0.025) and 320 [189 to 733] versus 265 [159 to 395] (*p* = 0.019), respectively), demonstrating the acute and systemic nature of the inflammatory process [43,44].

The ROC curves constructed for AISI, SII, and SIRI revealed excellent discriminative power, with AUC values of 0.825, 0.889, and 0.842, respectively—each exceeding the 0.8 threshold that defines strong diagnostic accuracy according to international standards. Among the three indices, SII demonstrated superior diagnostic performance, achieving a sensitivity of 79.53% and specificity of 86.31% at an optimal cut-off value of 975.75, as determined by Youden’s Index. These results surpass those of previous reports, where SII sensitivity rarely exceeded 75%, underscoring its enhanced predictive capability for differentiating gangrenous from chronic cholecystitis [1,30]. These indices should be regarded as complementary tools within the broader diagnostic framework, intended to refine rather than replace clinical judgment and imaging-based evaluation. In this retrospective analysis, their use in the preoperative setting may offer additional insight into disease severity and could potentially support surgical decision-making, particularly when interpreted in conjunction with established severity criteria such as those defined by the Tokyo Guidelines 2018 [5].

The AISI showed a cut-off value of 431.73, with sensitivity of 74.02%, specificity of 84.41%, and an overall accuracy of 81.03%, while SIRI, with a cut-off value of 1.49, achieved balanced sensitivity and specificity of 77.95%, corresponding to the same overall accuracy. These parameters reflect a robust and clinically meaningful diagnostic potential, particularly valuable in preoperative risk stratification [24,45].

By integrating cellular components that mirror both innate (neutrophil–monocyte) and adaptive (lymphocyte) immune responses, these indices offer a succinct representation of systemic inflammation and tissue injury. Although comprehensive scoring systems such as the Tokyo Guidelines Severity Score and APACHE II remain the standard for overall severity assessment, the present indices exhibit robust statistical performance (high AUC values and narrow confidence intervals) and practical utility as rapid, accessible adjuncts for early preoperative risk stratification [5,17,38].

### 4.3. Surgical Implications

The findings of this study also revealed a direct association between elevated inflammatory indices and intraoperative difficulty. In the GC group, the rate of conversion to open surgery was significantly higher (5.55% vs. 3.1%; *p* = 0.029), while the need for primary open cholecystectomy was nearly double (10.5% vs. 4.2%; *p* = 0.029). These observations are consistent with the existing literature, which identifies severe inflammation and gallbladder wall edema as major predictors of difficult dissection and increased risk of bile duct injury [38,41,46].

The preoperative assessment of composite hematological indices may therefore provide valuable information for surgical risk stratification and operative planning, helping to anticipate technical challenges, minimize conversion rates, and reduce postoperative complications [30,44,47].

ROC curve analysis further substantiates the clinical applicability of these markers, showing that an SII value above 975.75 and an SIRI value exceeding 1.49 can reliably identify patients at increased risk of complex or technically demanding dissection. Incorporating these indices into preoperative evaluation may help refine decision-making regarding surgical timing, operator expertise, and procedural approach, in alignment with the risk stratification principles outlined in the Tokyo Guidelines 2018 [1,5,10,38].

In this context, AISI, SII, and SIRI emerge not only as diagnostic tools but also as valuable predictors of technical complexity, capable of guiding a more personalized surgical strategy. In clinical practice, these indices can assist surgeons in stratifying preoperative risk. Patients presenting with markedly elevated inflammatory markers—specifically SII > 975.75, SIRI > 1.49, or AISI > 431.73—should be considered at high risk for gangrenous evolution and managed with expedited surgical intervention, multidisciplinary assessment, and intraoperative readiness for conversion. Integrating these thresholds with clinical signs (fever, vomiting) and imaging features (wall thickening, pericholecystic fluid) may offer a simple and reproducible approach for the early identification of severe disease and for informing surgical planning.

### 4.4. Study Limitations

This study should be interpreted in light of several inherent limitations. Its retrospective and single-center design restricts causal inference and may not fully capture the variability of clinical practice across different institutions. As a retrospective study, certain clinical confounders such as metabolic comorbidities, symptom duration, and imaging severity could not be uniformly retrieved, and were therefore not included in the regression models. The surgeries were performed by multiple surgeons with varying technical experience, a factor that could have subtly influenced the rate of conversion to open surgery and the subjective perception of intraoperative complexity. Additionally, heterogeneity in perioperative management and postoperative monitoring might have introduced minor, unavoidable differences in inflammatory response. Despite these aspects, the study benefits from a large, well-characterized cohort, uniform diagnostic criteria, and standardized laboratory assessment, which together enhance the reliability of the results. Future prospective, multicentric studies are warranted to validate these findings, refine the proposed diagnostic thresholds, and explore the dynamic behavior of these indices in the postoperative period.

## 5. Conclusions

The present study demonstrates that the inflammation-based indices AISI, SII, and SIRI serve as powerful, independent predictors of disease severity and surgical complexity in gangrenous calculous cholecystitis. Their strong diagnostic accuracy and correlation with clinical presentation, systemic inflammation, and intraoperative difficulty emphasize their potential as accessible, reproducible, and objective tools for early risk assessment.

By integrating these indices into the preoperative evaluation workflow, clinicians may enhance the early identification of gangrenous evolution, optimize surgical timing and team allocation, and ultimately improve patient safety and postoperative outcomes. Future prospective and multicentric validation studies could further consolidate their role as standard components of the diagnostic and prognostic algorithm in biliary inflammatory disease.

## Figures and Tables

**Table 1:** Demographic aspects.

Characteristic	All, *n* = 435	GC, *n* = 145	CC, *n* = 290	*p*
Age (M ± SD), years	62.34 ± 13.06	62.26 ± 13.06	62.38 ± 13.02	0.932
Gender, men	192 (44.1%)	64 (44.1%)	128 (44.1%)	1
Rural	200 (46%)	63 (43.4%)	137 (47.2%)	0.454

M = mean; SD = Standard Deviation; GC = gangrenous cholecystitis; CC = chronic calculous cholecystitis.

**Table 2:** Signs and symptoms.

Characteristic	All, *n* = 435	GC, *n* = 145	CC, *n* = 290	*p*
Abdominal pain	415 (95.4%)	144 (99.3%)	271 (93.4%)	0.006
Nausea	323 (74.3%)	121 (83.4%)	202 (67.7%)	0.002
Vomiting	241 (55.4%)	100 (69%)	141 (48.6%)	<0.001
Fever	37 (8.5%)	24 (16.6%)	13 (4.5%)	<0.001

GC = gangrenous cholecystitis; CC = chronic calculous cholecystitis.

**Table 3:** Variation in Inflammatory Markers.

Marker	All, *n* = 435	GC, *n* = 145	CC, *n* = 290	*p*
Lymphocytes (×10^9^/L)	1.80 [1.40 to 2.35]	1.52 [1.03 to 2.01]	1.94 [1.5 to 2.46]	<0.001
Monocytes (×10^9^/L)	0.44 [0.30 to 0.67]	0.48 [0.25 to 0.91]	0.43 [0.3 to 0.6]	<0.001
Platelets (×10^9^/L)	248 [207 to 304]	254 [204 to 315]	246 [207 to 297]	0.042
Neutrophils (×10^9^/L)	4.84 [3.68 to 7.88]	9.46 [6.60 to 13.4]	4.18 [3.29 to 5.17]	<0.001
AISI	300 [182 to 653]	826 [398 to 1817]	247 [151 to 350]	<0.001
SII	687 [445 to 1434]	1849 [1077 to 2731]	530 [392 to 743]	<0.001
SIRI	1.24 [0.75 to 2.47]	3.03 [1.55 to 7.14]	0.94 [0.62 to 1.41]	<0.001

GC = gangrenous cholecystitis; CC = chronic calculous cholecystitis; Data are reported as median [Q1 to Q3], where Q is the quartile. Mann–Whitney test was used to compare the two independent groups.

**Table 4:** Significant variation in inflammatory markers in patients with nausea.

Marker	All, *n* = 435	Nausea Present, *n* = 323	Nausea Absent, *n* = 112	*p*
Monocytes (×10^9^/L)	0.44 [0.30 to 0.67]	0.45 [0.30 to 0.69]	0.43 [0.30 to 0.60]	0.021
Platelets (×10^9^/L)	248 [207 to 304]	252 [210 to 308]	243 [196 to 287]	0.039
Neutrophils (×10^9^/L)	4.84 [3.68 to 7.88]	5.11 [3.77 to 8.55]	4.29 [3.21 to 5.75]	0.012
AISI	300 [182 to 653]	320 [189 to 733]	265 [159 to 395]	0.019
SII	687 [445 to 1434]	738 [463 to 1616]	530 [401 to 924]	0.012
SIRI	1.24 [0.75 to 2.47]	1.29 [0.76 to 2.64]	1.11 [0.69 to 1.65]	0.025

Data are reported as median [Q1 to Q3], where Q is the quartile. Mann–Whitney test was used to compare the two independent groups.

**Table 5:** SIRI-Binary logistic regression.

Variables in the Equation							
		B	S.E.	Wald	df	Sig.	Exp (B)
Step 1 a	SIRI	0.681	0.100	46.734	1	0.000	1.976
	Constant	−2.180	0.218	100.096	1	0.000	0.113

a. Variable(s) entered on step 1: SIRI.

**Table 6:** SII-Binary logistic regression.

Variables in the Equation							
		B	S.E.	Wald	df	Sig.	Exp (B)
Step 1 a	SII	0.002	0.000	79.224	1	0.000	1.002
	Constant	−2.847	0.258	121.760	1	0.000	0.058

a. Variable(s) entered on step 1: SII.

**Table 7:** AISI-Binary logistic regression.

Variables in the Equation							
		B	S.E.	Wald	df	Sig.	Exp (B)
Step 1 a	AISI	0.002	0.000	38.633	1	0.000	1.002
	Constant	−1.816	0.188	93.362	1	0.000	0.163

a. Variable(s) entered on step 1: AISI.

**Table 8:** Area Under the Curve for AISI, SII, SIRI.

Area Under the Curve	
Test Result Variable(s)	Area
AISI	0.825
SII	0.889
SIRI	0.842

## Data Availability

The original contributions presented in the study are included in the article, further inquiries can be directed to the corresponding author.

## Abbreviations

The following abbreviations are used in this manuscript:

LYM	Lymphocytes
MON	Monocytes
NEU	Neutrophils
PLA	Platelets
SII	Systemic Immune-Inflammation Index
SIRI	Systemic Inflammation Response Index
AISI	Aggregate Index of Systemic Inflammation
GC	Gangrenous cholecystitis
CC	Chronic calculous cholecystitis
AUC	Area under the curve
ROC	Receiver operating characteristic
